# Acute Appendicitis in the Setting of Infectious Mononucleosis: A Case Report

**DOI:** 10.7759/cureus.61619

**Published:** 2024-06-03

**Authors:** Kaedon Buchmiller, Michael G Smith, Michael J Valentine, Kyle K Turner, Brent Pickett

**Affiliations:** 1 College of Osteopathic Medicine, Kansas City University, Kansas City, USA; 2 Department of Orthopaedics, Logan Regional Orthopedics, Intermountain Health, Logan, USA

**Keywords:** unusual presentation, cough, abdominal pain, epstein-barr virus, acute appendicitis, infectious mononucleosis

## Abstract

Infectious mononucleosis (IM) is a viral illness caused by the Epstein-Barr virus that typically manifests with pharyngitis, lymphadenopathy, and fatigue. In rare cases, IM can cause acute appendicitis. We present the case of an 18-year-old female who arrived at the emergency department with worsening abdominal pain and an ongoing cough. Initial imaging showed a questionably dilated appendix, and a follow-up examination revealed cervical lymphadenopathy. She later returned to the ED with severe abdominal pain, clinical signs of acute appendicitis, and a positive monospot test, which led to an appendectomy. This case illustrates the need for complete history taking and thorough physical examination in patients with acute appendicitis, as their condition may be due to an atypical underlying cause.

## Introduction

Infectious mononucleosis (IM) is a highly contagious condition caused by the Epstein-Barr virus (EBV) [1]. Typical symptoms include fever, malaise, and fatigue, followed by lymphadenopathy, tonsillitis, pharyngitis, and possibly splenomegaly, which may last up to a month [1]. Suspected cases of IM are confirmed using a heterophile antibody test, commonly known as a monospot test [2]. Abdominal pain is an uncommon symptom in patients with IM, and its presence may rarely be indicative of acute appendicitis [3].

The following report recounts the case of a young woman who presented to the emergency department (ED) with clinical features concerning for acute appendicitis. Initial imaging showed a questionably dilated appendix. A follow-up examination revealed tender cervical lymphadenopathy. She later returned to the ED with more severe right lower quadrant (RLQ) abdominal pain, and a diagnosis of IM was made. Due to worsening symptoms consistent with acute appendicitis, an appendectomy was performed.

## Case presentation

**Figure 1:**
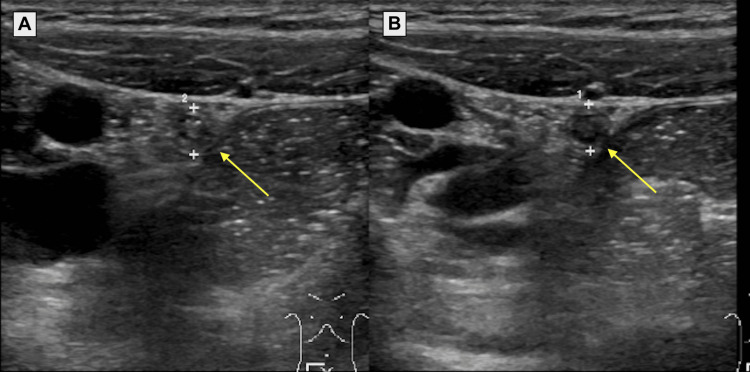
Ultrasonography of the appendix from the first (A) and second (B) emergency department visits. Panel A shows a questionably dilated appendix measuring 6.5 mm in diameter. Panel B shows a normal appendix measuring 6 mm in diameter with trace surrounding fluid. It is generally accepted that a normal appendix does not exceed 6 mm in diameter [4].

An 18-year-old female collegiate runner with no significant medical history initially presented to the emergency department with one week of intermittent right-sided abdominal pain. At the time of presentation, the patient also reported a persistent cough for the last two weeks, which was treated with augmentin. A review of systems was otherwise negative. Her physical examination was remarkable for mild reproducible tenderness without rebounding or guarding in the RLQ. Laboratory tests, including a complete blood count, basic metabolic panel, liver function tests, and lipase, were all within normal limits. Ultrasound showed a questionably dilated appendix at 6.5 mm in diameter (Figure 1A). A computed tomography (CT) scan of the abdomen and pelvis revealed a normal appendix, a left ovarian cyst, and mild right adnexal free fluid. Due to the imaging findings, the patient was referred to outpatient gynecology for evaluation of the ovarian cyst.

At the follow-up examination, the patient reported ongoing abdominal pain that worsened with movement. She also reported tender lumps under her right ear and on the right aspect of her occipital bone. Physical examination showed tender preauricular lymph nodes and a firm lump on the right occipital bone. Ultrasound confirmed the presence of a left ovarian cyst as well as mild right adnexal free fluid. She was counseled on symptomatic care of the ovarian cyst rupture and referred to ear, nose, and throat (ENT) specialists for evaluation of her lymphadenopathy.

Later that evening, the patient returned to the ED with abdominal pain worsening in severity and frequency. A pregnancy test was negative. Physical examination identified moderate tenderness at McBurney's point and guarding with rebound tenderness. She had a positive monospot test. Abdominal ultrasound showed a normal appendix at 6 mm with trace surrounding fluid (Figure 1B). Ultrasound of the neck showed multiple enlarged lymph nodes suspicious for malignancy. Given the clinical findings and the patient's concerns for appendicitis, surgery was consulted and an appendectomy was performed. At postoperative day 10, the patient was without major complaints. Her pain was well controlled and she had resumed most normal activities. Evaluation by ENT showed significant improvement in lymphadenopathy. A timeline of the patient’s acute infectious course is found in Table 1.

**Table 1:** A timeline of the patient's acute infectious course, including clinical findings, diagnostic and imaging studies, and treatment. The timeline begins with the patient's initial self-reported symptoms. ED: Emergency Department; CBC: complete blood count; BMP: basic metabolic panel; LFTs: liver function tests; US: ultrasound; CT: computed tomography; ENT: ear, nose, and throat.

Day	Clinical Findings	Diagnostics and Imaging	Treatment
Day 0-13	Non-productive cough, mild abdominal pain		Prescribed Augmentin for cough
Day 14	Intermittent, right-sided abdominal pain and persistent cough; evaluated in ED	CBC, BMP, LFTs, and lipase were all within normal limits; US revealed a mildly dilated appendix; CT revealed a normal appendix, left ovarian cyst, mild right adnexal free fluid	Referred to outpatient gynecology for evaluation of ruptured ovarian cyst
Day 15 (Morning)	Persistent abdominal pain, tender preauricular lymph nodes, and a firm lump on the right occipital bone	Transvaginal US confirmed left ovarian cyst and mild right adnexal free fluid	Counseled on symptomatic care of ruptured ovarian cyst and referred to ENT for evaluation of lymph nodes
Day 15 (Evening)	Severe abdominal pain, moderate tenderness at McBurney's point with guarding and rebound tenderness; evaluated in ED	Positive monospot test; negative pregnancy test; appendix US revealed trace surrounding fluid; neck US revealed bilateral cervical and posterior auricular lymphadenopathy	Surgery was consulted and appendectomy was performed
Day 25	Resolution of abdominal pain, lymphadenopathy, and cough		Resume normal activities as tolerated

## Discussion

In the United States, the incidence of IM is 5/1000 per year [5]. IM is often referred to as "mono" or the "kissing disease" due to its spread via bodily secretions, especially saliva [6]. Early symptoms of IM typically include fever, malaise, and fatigue, whereas later manifestations may consist of acute pharyngitis, tonsillitis, lymphadenopathy, and splenomegaly [1]. Symptoms typically occur in adolescents and young adults and last 2-4 weeks [1]. Suspected cases of IM are confirmed with a positive monospot test. When clinical suspicion of IM is high and a monospot test is negative, serology may be performed to identify anti-EBV nuclear antigen-antibodies (EBNA), which develop 6-12 weeks after the start of primary infection and remain detectable for life [2]. Approximately 90-95% of adults are EBV-seropositive worldwide [7]. Treatment of IM is mainly symptomatic (e.g., fluids, analgesics, antipyretics) as there is no currently accepted specific treatment for IM [1]. Splenomegaly is often a complication of IM, and physical activity should be avoided for 21 days after the initial symptoms present to avoid the risk of splenic rupture [8].

Documented cases of acute appendicitis in patients with IM are rare. Abdominal pain was the most frequently reported symptom in these cases. Notably, the majority of affected patients exhibited other characteristic symptoms of IM, either before or concurrent with presentation.

In one report, a 19-year-old female presented with two days of abdominal pain in the RLQ associated with vomiting, nausea, and anorexia. A CT scan revealed early appendicitis and an appendectomy was performed. Three days after her surgery, the patient developed multiple fever spikes unresponsive to antibiotics. The diagnosis of IM was made after EBV serology revealed high EBV antibody titers [3].

In another report, a 15-year-old female presented with two days of abdominal pain in the lower quadrants, vomiting, and diarrhea. Physical exam revealed guarding and rebound tenderness of the abdomen, as well as bilateral cervical lymphadenopathy. A monospot test was positive. An appendectomy was performed [9].

Appendicitis, although a rare complication of IM, is a recognized consequence that can pose diagnostic challenges. The clinical presentation is often dominated by symptoms of appendicitis, which can complicate the identification of the underlying IM [3]. Lymphoid follicles are found within the submucosa and lamina propria of the appendiceal wall [10]. EBV-infected lymph nodes show reactive follicular hyperplasia due to increased activation of B lymphocytes [11]. Acute appendicitis in IM is thought to arise from the obstruction of the appendiceal lumen, which occurs due to the swelling of lymphoid tissue during the acute phase of IM and leads to the classic presentation of acute appendicitis [12].

## Conclusions

Infectious mononucleosis can manifest with atypical complications, including acute appendicitis. In rare cases, acute appendicitis may be the initial presentation of IM. Therefore, when a patient presents with clinical symptoms suggestive of appendicitis, it is crucial to conduct a comprehensive evaluation, including a detailed history, and consider IM as a potential underlying etiology. Accurate diagnosis necessitates close clinical, laboratory, and radiological monitoring of these patients.
